# Emerging Perspectives in the Formulation of Lyophilized Orally Disintegrating Tablets: From Lyoc to Self-Nanoemulsifying Lyophilized Tablets (SNELTs) and Beyond into Hybrid Platforms

**DOI:** 10.3390/pharmaceutics18050615

**Published:** 2026-05-18

**Authors:** Eliza Grațiela Popa, Liliana Mititelu Tartau, Alina Diana Panainte, Larisa Păduraru, Andreea Crețeanu

**Affiliations:** 1 Faculty of Pharmacy, Grigore T. Popa University of Medicine and Pharmacy Iasi, 16 Universitatii Street, 700116 Iasi, Romania; eliza.popa@umfiasi.ro (E.G.P.); larisa-paduraru@umfiasi.ro (L.P.); andreea.creteanu@umfiasi.ro (A.C.); 2 Faculty of Medicine, Grigore T. Popa University of Medicine and Pharmacy Iasi, 16 Universitatii Street, 700116 Iasi, Romania; liliana.tartau@umfiasi.ro

**Keywords:** lyophilization, ODTs, SNELTs, hybrid platforms, 3D printing

## Abstract

Four decades have elapsed since orally disintegrating tablets (ODTs) were first formulated as the emulsion/type Lyoc tablet, a porous mass intended to rapidly disperse in saliva. Following the lyophilization process, new formulations of ODTs were designed, intending to make a simpler and more reproducible formulationZydis, LBL-Flash, Quicksolv, and, more recently, Zydis Ultra. Lyophilization is widely recognized as an effective technique for the development of ODTs, due to its ability to produce highly porous structures that enable rapid disintegration and improved patient compliance. However, its advantages should be considered in relation to other manufacturing methods, as each technology presents specific trade-offs in terms of cost, scalability, mechanical strength, drug loading capacity, and process robustness. In line with the modern sustainable and green pharmacy trend, new raw materials have gained attention as excipients for lyophilized ODTs; these materials include certain plant derivatives, but also performant excipients with newly discovered functionalities. At present, a new generation of ODTs is available in the form of Self-Nanoemulsifying Lyophilized Tablets (SNELTs), which bring the advantages of Self-Nanoemulsifying Drug Delivery Systems (SNEDDS) into ODTs via the lyophilization method. The technique is mostly applicable to low-solubility drugs formulated as nanoemulsions, which are absorbed onto solid carriers and further lyophilized, forming the final ODT. Despite its limitations (expensive, time-consuming, and high product friability), lyophilization is being continuously developed nowadays, in combination with other techniques (3D printing, mucoadhesion, or electrospinning), building hybrid platforms for the modern ODTs of the future.

## 1. Introduction

In 1985, the French company L. Lafon formulated the first oral lyophilisates, with model drugs phloroglucinol and paracetamol, publishing the patent by the name *Lyoc* (1986) [1,2]. This product consisted of a porous mass obtained from an oil-in-water emulsion with rapid dispersion in saliva (within seconds), without the need for water [1,2,3]. Regardless of the complexity of the formulation, the elaborate and long lyophilization process, and the sensitivity of the final product, this design gained attention from the pharmaceutical world. Rapid release of the drugs, the possibility of bioavailability increase, and the advantages for certain categories of patients (elderly, children) led to the emergence of a new dosage form: *orodispersible tablets* or *orally disintegrating tablets (ODTs)* [4,5].

Consequently, the French Pharmacopoeia included a monograph for oral lyophilisates, and, later on, the European Pharmacopoeia included the orodispersible tablets as a sub-chapter in the monograph entitled *Tablets* [4,5,6]. The compendial definition is ‘uncoated tablets intended to be placed in the mouth, where they disperse rapidly before being swallowed’, with the disintegration condition as follows: ‘orodispersible tablets disintegrate within 3 min’ [6]. The FDA condition, however, limits the ODT disintegration time to ‘approximately 30 s or less’, when based on the United States Pharmacopeia (USP) disintegration test method or alternative [7].

Following the lyophilization process, new formulations of orally disintegrating tablets were designed, intending to make a simpler and more reproducible formulation than the emulsion/type Lyoc tablet. Thus, in 1986, the Scherer company (now a Catalent division) patented the *Zydis* formulation, consisting of a freeze-dried viscous suspension, with the possibility of implementing the technology on a larger industrial scale [8,9]. Other formulations with varying compositions, but still using lyophilization as the production method, were designed: *Pharmafreeze*, *Quicksolv*, *LBL-Flash*, etc. [10,11,12]. Given their advantages, in the following years, the pharmaceutical companies put ODTs in the spotlight, researching and finding ways to manufacture them by conventional methods while at the same time keeping their optimal characteristics. This was not an easy task, given the ideal desired features for ODTs: (a) disintegrates in the mouth within seconds; (b) allows formulation with high doses of drugs; (c) can be taste-masked and has no residual taste; (d) has minimum or zero grittiness upon dispersion in saliva; (e) has adequate hardness, which allows transportation and manipulation without breakage; (f) is not sensitive to environmental conditions (temperature, humidity); and (g) can be manufactured with conventional equipment and low costs [13,14,15].

Given the many advantages of ODTs, there are certain patient categories targeted for benefiting from this dosage form: patients suffering from acute or chronic pain; neurological patients (Parkinson’s disease, epilepsy, psychotic patients, sleep disorders, etc.); patients with allergies; immunotherapy; motion sickness; and also generally age categories such as geriatric and pediatric patients [14,15,16]. Consequently, the therapeutic classes of drugs that have been approached and widely formulated as ODTs are the ones which need rapid therapeutic effect: analgesics (paracetamol) [17,18]; NSAIDs (ibuprofen [19,20], piroxicam [21,22], nimesulide [23], rofecoxib [24,25], and valdecoxib [26]); neuroleptics (olanzapine [27], risperidone [28]); antihistaminic drugs (loratadine [29,30], cetirizine [31], desloratadine [32], and bilastine [33]); 5-HT_3_ inhibitors (ondansetron [34]); and 5-HT-1 agonists as anti-migraine drugs (sumatriptan [35], zolmitriptan [36]). The majority of these drugs have the advantage of being active in low doses, which mainly overcomes the formulation impediment of taste masking.

Other technologies emerged besides freeze-drying, and were used in obtaining orodispersible tablets, but none comply with all the targeted ideal features. Direct compression with or without effervescent systems has been implemented with success, using processed polyols as fillers (mannitol, sorbitol, and lactitol) and superdisintegrants, in order to achieve fast disintegration (sodium starch glycolate, sodium crosscarmellose, and crosspovidone) [37,38]. *Orasolv* and then *Durasolv* orodispersible tablets met most of the preferred characteristics; nevertheless, due to the direct compression method, their disintegration time lies in the range of 30–50 s [39,40]. Other particular methods have been tested in terms of obtaining different types of ODTs over time, such as wet granulation in a fluid bed with processed fillers, molding (wet compression), melt granulation, and sublimation [15,17,41,42].

Lyophilization (freeze-drying) is a special technique involving the evaporation of ice at low pressures and temperatures by the use of special equipment (lyophilizers). Applied to the manufacturing of ODTs, this leads to highly porous structures, also called *oral lyophilisates*, through the sublimation of water from the frozen drug–polymer matrix, resulting in remarkably rapid disintegration due to the capillary action inside the sponge-like structure. Conversely, in a tableting process, structural integrity is attained through direct compression of the powder to form the bonds between the particles [43,44].

The mixture to be freeze-dried has to be formulated as a thick suspension with a pasty consistency, so that after evaporation, a porous mass results, that will quickly absorb water and disintegrate.

Compared to all the other methods of obtaining ODTs, lyophilization imparts excellent qualities to the final product: the disintegration time is less than 15 s, the mouthfeel of oral lyophilisates is more pleasant than that of compressed ODTs, which, regardless of the new fillers with higher degree of fineness and potent superdisintegrants used in the formulations, have more grittiness and longer disintegration times, due to a decrease in tablet porosity during compression. Also, the mass and content uniformity are usually more reproducible in the lyophilization method, since it involves pouring of a viscous product (liquid, paste) into blister pack shells, compared to the homogeneity issues that can impact the direct compression method (Table 1) [45,46]. Numerous studies indicate higher performance regarding the dissolution and bioavailability of the incorporated drugs compared to other methods. However, the highly porous structure of lyophilized ODTs also increases their sensitivity to environmental moisture during storage and transportation. Therefore, these formulations generally require protective moisture-resistant packaging systems, such as peel-off or Alu-Alu blister packs, in order to maintain their physical stability and rapid disintegration properties. Additional preventive strategies include the incorporation of moisture-protective excipients, optimization of residual moisture content after lyophilization, and storage under controlled temperature and humidity conditions.

From a formulation perspective, the use of carefully selected cryoprotectants and bulking agents (e.g., sugars such as trehalose or mannitol) can significantly improve cake structure, prevent collapse, and enhance the stability of both the active pharmaceutical ingredient and the final orally disintegrating tablet (ODT) [13,14,15,16]. In addition, the development of multifunctional excipients, including co-processed and plant-derived materials, contributes to improved mechanical strength, faster disintegration, and better process robustness. [16,46].

From a process standpoint, the optimization of freezing protocols (controlled nucleation, annealing steps) and primary/secondary drying parameters can markedly reduce drying time while improving batch uniformity and product quality. Advances in freeze-dryer design, including improved heat transfer systems and real-time process monitoring via Process Analytical Technology (PAT) tools, also enhance reproducibility and scalability. Furthermore, continuous manufacturing approaches and cycle optimization strategies are increasingly being explored to reduce production costs and improve industrial feasibility [43,44,45,46].

Finally, emerging technological solutions, as well as hybrid systems combining lyophilization with alternative techniques, are being investigated to overcome inherent limitations of conventional freeze-drying. Taken together, these strategies indicate that while lyophilization has intrinsic constraints, many of them can be mitigated through integrated formulation and process innovations, thereby supporting its continued relevance in the development of high-performance ODT systems.

## 2. Search Strategy

This narrative review was conducted to summarize current evidence regarding the development, formulation, and recent advances in lyophilized orally disintegrating tablets and related drug delivery systems. A comprehensive literature search was performed using the following electronic databases: PubMed/MEDLINE, Google Scholar, Scopus, and Web of Science.

The search covered articles published between January 2015 and March 2026, with particular emphasis on studies published after 2020 in order to capture the most recent scientific developments in the field. The search strategy combined Medical Subject Headings (MeSH) terms and free-text keywords, including but not limited to the following: lyophilization, freeze-drying, orally disintegrating tablets, ODTs, self-nanoemulsifying drug delivery systems, SNEDDS, SNELTs, and pharmaceutical excipients. Boolean operators (AND, OR) were used to refine and optimize the search results. Eligible studies included original research articles, systematic reviews, and meta-analyses addressing formulation strategies, technological advancements, and the performance evaluation of lyophilized ODT systems.

Only articles published in English were considered for inclusion. Study selection was based on relevance to the topic, scientific quality, and recency of publication. In addition, the reference lists of selected articles were manually screened to identify further relevant studies that may not have been captured in the initial database search.

No formal systematic review protocol was registered, and no quantitative synthesis (meta-analysis) was performed, as the aim of this work was to provide a qualitative and narrative overview of the field. Given the narrative nature of the review, strict inclusion and exclusion criteria were not applied; however, priority was given to peer-reviewed studies, high-impact publications, and recent advances contributing to the understanding of lyophilized ODT technologies. The review was conducted in accordance with general principles of transparency and reproducibility for narrative reviews.

## 3. Types of Freeze-Drying ODT Technologies

### 3.1. Lyoc

This was the first oral lyophilisate product on the market, providing short disintegration times and the incorporation of high-dose drugs, but with the limitations of low hardness and high friability. The product is based on the freeze-drying of an oil/in/water (O/W) emulsion, more or less viscous, which forms what is called a *Lyoc-paste* type of product [1].

The lipophilic phase of the emulsion is an oil that contains preferably triglycerides of the C_8_–C_10_ fatty acids, such as cotton seed oil, soy oil, sesame oil, peanut oil. The aqueous phase is the one that will form the tablet matrix, containing polyols or other hydrophilic carbohydrate derivatives (e.g., mannitol, maltodextrin, milk powder, etc.) and a viscosity agent (acacia, tragacanth, xanthan gum, pectin, cellulose derivatives, polyvinyl alcohol). The surfactants that form the O/W emulsion are added to both phases and may be nonionic surfactants such as polysorbates (Tween), sorbitan esters (Span), polyethoxylated castor oil, or lecithin from eggs or soybeans. Other excipients are added for taste masking and palatability enhancement: sweeteners such as sugar, glucose, sorbitol, but also saccharin, cyclamate or other additives (citric acid, tartaric acid, flavors). The drug is included in one of the phases of the emulsion during preparation, or in the final emulsion, upon stirring [1,3,47].

Another suspension-type Lyoc formulation has also been described, which is called *Lyoc-sorbet*, allowing the incorporation of active drugs at low temperatures (0 °C), by a technique similar to that employed in the preparation of food-grade ice cream. The suspending agents are hydrophilic polymers which prevent drug sedimentation, but, at the same time, used in high amount, may prolong the disintegration time of the final ODTs. The advantage of the suspension-type Lyoc is that it can incorporate high doses of drugs (up to 700 mg) [1,3,48].

### 3.2. Zydis

Zydis is a technology that emerged shortly after the Lyoc lyophilisate, with the aim of overcoming the difficulties linked to the preparation of the emulsion-type ODT. It was first patented in the 1970s, in England, with the name *Expidet*, which then was reformulated as *Zydis* and described in the American patent no. 5,837,287/1988, for the drugs domperidone and loperamide [49]. The formulation is based on a freeze-dried suspension containing saccharides (polyols) and soluble polymers (gelatin, alginates) as carriers for the dispersed drug [8,9]. The saccharide/polymer ratio is carefully optimized in order to provide rapid disintegration of the final product; the formulation usually contains other adjuvants, such as the following: wetting agents, protective agents (e.g., glycine), flocculating agents (e.g., xanthan gum) preservatives, antioxidants, colorants, flavors, pH regulators [5,8,9]. The Zydis technology has been adapted for industrial scale, with the following manufacturing steps [5,50]:

- The drug is dissolved or dispersed in an aqueous solution containing the carrier and the saccharide.
- The mixture is poured in the blister pack alveoli, avoiding sedimentation; the alveoli are placed on trays.
- The trays are passed through a liquid nitrogen tunnel, where the freezing step takes place; the frozen product is stored in the refrigerator for a certain time, while the amorphous substances may crystallize, increasing product stability.
- The trays are put in a lyophilizator, where the sublimation step takes place.
- After the quality control step of the finished product, the alveoli are sealed with a special foil that protects the ODTs from moisture: polyvinyl chloride, polyvinylidene chloride, etc.

The Zydis technology has been implemented in the formulation of over 35 products reported by the Catalent Company who owns the patent, some of the examples are presented Table 2.

*Zydis Ultra* is a new generation of Zydis technology, which has been patented by the Catalent company in order to overcome the limitations of the classic Zydis ODT. In Zydis Ultra, the drug can be loaded in higher doses (up to 50% of the total product mass, up to 400 mg) and usually involves taste masking techniques, by coating drug particles with a thin polymer film (the *Zydis Ultra-Coating Technology*) [9,16].

### 3.3. Pharmafreeze

This technology was first described in the U.S. Patent no. 5,298,261/1994 and is based on lyophilization, but slightly modified vs. the basic technique [10]. The ODTs prepared through this technology may have drug content up to 60% and a dose of 1–250 mg/tablet; other ingredients are a matrix former polymer (xanthan gum, carrageenan 0.1–0.3%), a polyol (mannitol 10–60%), a surfactant (polysorbate, max. 1%), flavors and sweeteners. The lyophilization process is done in two steps: (a) freezing a viscous mixture containing the drug (paste, thick suspension) and (b) primary drying under vacuum, which evaporates the water above the collapse point of the matrix, but below the eutectic point. In such conditions, above the collapse point, the matrix maintains a high mobility, the dissolved particles of carbohydrate are joined, and the unbound water evaporates due to partial liquefying. By comparison with other technologies, the matrix undergoes a partial collapse of its network, is lyophilized in standard conditions and therefore results in a product with a denser structure, and smaller pores [10,50,51].

### 3.4. Quicksolv

This is a special lyophilization technique, in which the frozen matrix containing the drug, including the matrix-forming agent (gelatin), a polyol (mannitol), a viscosity agent (carbomer), surfactants and sweeteners, is immersed for 5 h in anhydrous alcohol at a temperature of −15 °C, for the removal of water. When the ice has been totally dissolved in ethanol, the product is further transferred to drying upon vacuum for 1 h, in order to totally evaporate the residual ethanol. The final product is an ODT with very short disintegration time, but the method is applicable only to the drugs that are not soluble in the extraction solvent and is also more appropriate for low-dose drugs [11,50]. The Quicksolv technology has been applied in the formulation of ODTs with domperidone, risperidone and cisapride (Table 2).

**Table 2:** Examples of drugs formulated as ODTs through lyophilization [9,52].

Marketed Product	ODT Technology
Olanzapin (Zyprexa^®^)	Zydis
Loratadine (Claritine^®^ Reditabs)	Zydis
Ondansetron (Zofran^®^)	Zydis
Rizatriptan (Maxalt^®^ MLT)	Zydis
Loperamid (Imodium^®^ Lingual)	Zydis
Rimegepant (Nurtec^®^ ODT)	Zydis
Famotidine (Pepcid^®^)	Zydis
Phloroglucinol (Spasfon^®^)	Lyoc
Metopimazine (Vogalene^®^)	Lyoc
Piroxicam (Feldene^®^)	Lyoc
Carvedilol (Dilatrend^®^)	Lyoc
Cisaprid (Propulsid^®^)	Quicksolv
Risperidone (Risperdal-M-Tab^®^)	Quicksolv
Domperidone (Motilium^®^)	Quicksolv

### 3.5. Self-Nanoemulsifying Lyophilized Tablets (SNELTs)

The lyophilization of micro/nano encapsulated drugs has been reported since 2004 in the U.S. Patent 6,833,192, describing the technology called *LBL-Flash* [12]. This is an ODT technology which utilizes a special layering technique which involves forming polymer layers (e.g., chitosan, dextran) around the drug particles by electrostatic interaction, as dense and arranged polyelectrolytic complexes. The final drug-loaded capsule has a thickness of 8–50 nm, with four to 20 layers. The LBL-Flash technology has been recommended for low-soluble and insoluble drugs, with the formed drug-loaded nanocapsules being further formulated for lyophilization. Thus, the obtained product would provide high drug dissolution, given the increased specific surface upon dispersion in saliva; also, the electric charge of the particles is supposed to maintain the stability of the suspension during the lyophilization process. This technique was also described in the European Patent in WO2004030648, being implemented for two BCS class II drugs: carbamazepine (25 mg) and ketoprofen (25 mg) [53].

A preliminary form of lyophilized emulsions had been formulated since 1998, by freeze-drying oil-in-water emulsions, with the aim of increasing drug bioavailability. Upon lyophilization of these macroemulsions, ODTs were formed, named LDE (lyophilized dry emulsions) tablets [54].

**Figure 1:**
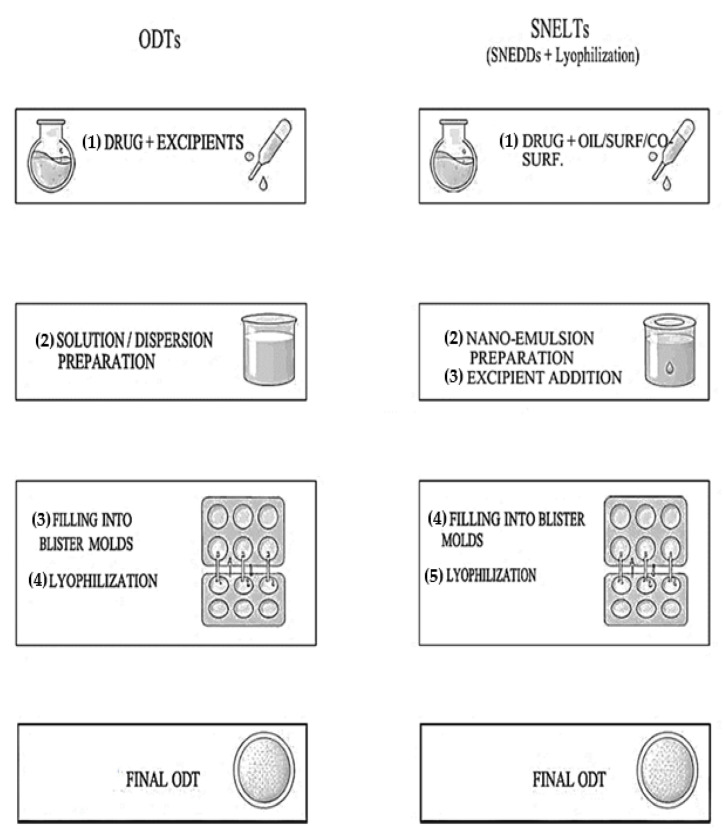
ODTs vs. SNELTs through the lyophilization method. (Used Ai-Claude Opus 4.5).

At present, a new generation of orodispersible tablets are the Self-Nanoemulsifying Lyophilized Tablets (SNELTs), which bring the advantages of Self-Nanoemulsifying Drug Delivery Systems (SNEDDS) into orally disintegrating tablets, via the lyophilization method. The technique is mostly applicable for low-soluble drugs (BCS classes II and IV), formulated as a nanoemulsions, which are adsorbed onto solid carriers and further freeze-dried, to form the final ODT. The formulation of SNELTs involves a two-step optimization: (a) formulation and preparation of nanoparticles (nanoglobules) with good stability, loading drug as SNEDDS and (b) formulation and preparation of freeze-dried ODTs, by mixing matrix-forming excipients and other additives with the given drug-loaded nanoparticles [55] (Figure 1). The SNEDDS formulation involves the preparation of an isotropic mixture of the drug, an oily phase, a surfactant, and a co-surfactant, which would spontaneously form a nanoemulsion when gently dispersed in water [21,55].

This newly emerging type of oral lyophilisates, still in its pioneering era, aims at transmucosal delivery and absorption of poorly soluble drugs. It has been achieved for vitamin K, a lipophilic drug with a poor bioavailability (ranging from 10 to 63% depending on the formulation), by preparation of SNEDDS using Transcutol and Labrafac as surfactants. The optimized nanoglobules showed good stability and could be freeze-dried in order to enhance vitamin K bioavailability [56].

The literature data report a new research direction in combinations of APIs with essential oils for complementary action on various pathologies [57,58]. Sindi et al. studied a meloxicam–peppermint oil combination loaded into SNEDDS for the management of periodontal pain. The obtained self-nanoemulsion lyophilized composites (SNELCs) aimed at enhancing meloxicam solubility and preventing the gastric irritation of the anti-inflammatory drug by adding various proportions of peppermint oil. The very short disintegration time (~3 s) of the lyophilisates may be related to the surfactants used in the formulation of the nanoglobules. The in vitro dissolution test showed a 50% release of meloxicam within the first 5 min, with a 91% release after 1 h [58].

In line with the advances in transmucosal delivery of low-soluble drugs, the anti-gout drug febuxostat was formulated in SNEDDS, optimized by varying the oily phase (castor oil/clove oil/oleic acid/linoleic acid), the surfactants (polyethylene glycol 40-stearate/Kolliphor/Tween 80/Tween 20/Labrafac) and the co-surfactants (PEG 4000/PEG 200/Transcutol). Consequently, the obtained nanoemulsions were lyophilized into SNELTs, which could be optimized by varying the proportions of the matrix formers (gelatin, HPMC) and superdisintegrant (croscarmellose) [59].

SNELTs have been recently formulated for drugs with the rapid onset of action, such as the sedative-hypnotic zaleplon combined with lavender oil, resulting in orodispersible tablets with disintegration times within 30 s and a 17 times higher in vitro release rate of zaleplon compared to the immediate release commercial product containing the same drug [57].

A type of nanoparticle loaded with rivaroxaban has been studied by El-Hady et al. as nano-mixed micelles (NMMs), with a size below 200 nm. The NMMs were optimized using various surfactants and further mixed with excipients for the lyophilization step: sodium alginate–gellan gum mixture, low acyl gellan gum alone and hydroxypropyl methylcellulose. The results showed that the combination of sodium alginate/gellan gum met the conditions for in vitro disintegration time, drug release (over 80% within 90 min), acceptable friability. The in vivo disintegration time was also tested on volunteers, with shorter times than the in vitro results (31.5 to 82.5 s) [60].

A study involving the 5α-reductase inhibitor finasteride (a BCS class II drug) was performed by Ahmed et al., in order to enhance its solubility and bioavailability, by formulating into SNELTs, with an optimization according to a Box–Behnken design of experiments [55]. The prepared SNEDDS were mixed with matrix former excipients (HPMC and Plasdone XL) and further lyophilized. By comparison with the marketed tablets containing finasteride, an increase in drug release and in vivo bioavailability was noticed; however, regarding the disintegration, the tablets disintegrated in more than 3 min [55]. This could be attributed to the binding action of HPMC, but also to the crosslinking between the strands of gelatin during the freeze-drying process, leading to the formation of a three-dimensional gel network matrix, increasing tablet hardness [55].

In other approaches, freeze-drying is used as an intermediate phase for optimizing the SNEDDS, in order to increase drug solubility and mask the taste of the drug. This has been implemented for cyclosporine: the SNEDDS were adsorbed onto amorphous silica, mixed with water and the obtained slurry was lyophilized. Then, the lyophilized product was mixed with superdisintegrant and fillers and further compressed to obtain orodispersible tablets [61].

Out of all the possibilities of formulation for bioavailability enhancement, nanoemulsions as SNELTs provide the highest stability, due to loading the drug into nanoglobules, combined with freeze-drying, which protects the drug, but also other excipients, such as flavors, from thermal degradation [56]. The reported studies showed that SNELT particle size is significantly influenced by the oil/surfactant/co-surfactant ratio. Globules with a mean size below 200 nm meet the criteria for stable systems to be lyophilized as SNELTs [59].

In addition, incorporation of the API into SNEDDS nanoglobules may protect poorly soluble or sensitive drugs from aggregation and chemical degradation, while the lyophilization process removes water from the system, thereby reducing hydrolytic degradation and improving long-term stability during storage. The presence of surfactants and co-surfactants in the formulation of nanoglobules loading the drugs poses tolerance issues, which can be overcome by choosing the right proportions of well-tolerated derivatives, such as Transcutol (diethylene glycol monoethyl ether) or Labrasol (caprylocaproyl polyoxyl-8 glycerides) [58,59]. The addition of mannitol or other polyols as bulking agents is preferred, since it provides adequate mechanical strength to the final lyophilisates [56,57,58].

Table 3 shows by comparison the main advantages and limitations of the lyophilization technologies involved in ODT preparation.

## 4. Excipients in the Formulation of Freeze-Dried ODTs: From Past to Present

The main categories of excipients used in the formulation of lyophilized ODTs are [4,5,50,51]:

- Matrix formers provide the cohesion of the matrix, increase tablet strength and decrease tablet friability;
- Bulking agents (porosity enhancers) build the ‘skeleton’ of the lyophilisate, increase porosity, accelerate disintegration and also improve palatability;
- Cryoprotectants protect the sensitive drugs in the freezing steps;
- Collapse protectants maintain matrix porosity during the primary drying step;
- Palatability enhancers (sweeteners, flavors).

One important direction in the formulation of freeze-dried ODTs focuses on new ingredients that are meant to overcome the limitations of traditional excipients (gelatin or high concentrations of saccharides), in order to improve stability, disintegration speed, and compatibility for diabetic patients. Also, the need for sustainable raw materials has opened new directions for using plant derivatives as binders or disintegrants in the formulation of ODTs, obtained first through compression methods, and over the latest years being translated and implemented also into the lyophilization method as well.

The most researched categories have been the matrix formers and bulking agents, since they make up the majority of the tablet mass. Table 4 displays examples of the traditional and emerging excipients as matrix formers/bulking agents in the lyophilization method for ODTs.

A new line of research has been focusing in the past years on zero-saccharide ODT formulation, by replacing the saccharide/polyols with amino acids [62]. The following section summarizes ‘old and new’ main excipients used in freeze-dried ODT formulation.

### 4.1. Gelatin

Gelatin is a scleroprotein extracted from animal tissues containing collagen (pig, calf, fish) by acid hydrolysis (type A) or alkaline hydrolysis (type B). Commercial grades are usually a mixture of both types; this is why the name ‘gelatin’ covers a range of products having different properties. The jellification capacity of gelatin is expressed as Bloom strength, varying from 100 to 200 in pharmaceutical applications [63].

In ODTs prepared by lyophilization, gelatin is a good matrix former and mostly used, as reported by the literature, being still considered the ‘golden standard’ [50,51,64,65] (Table 5). For the preparation of the pasty, mixture gelatin is dissolved in water usually in a concentration of 2–5% and is mixed with other fillers (cellulose derivatives, polyols, etc.). It has been demonstrated that increasing gelatin concentration leads to a higher network strength upon lyophilization, which leads consequently to longer disintegration times and higher hardness (expressed as fracturability, measured in Newtons) [64].

Higher gelatin concentrations (14%) result in a disintegration time within 1 min, whereas concentrations of 20–22% gelatin lead to disintegration times that exceed 3 min [66]. The gelatin concentration will be chosen depending on the other ingredients used in the formulation (gums, cellulose derivatives), but generally a concentration below 1% tends to form friable ODTs, with very low hardness [67]. Because of its animal origin, there are ethical issues regarding gelatin use in pharmaceuticals; therefore, in hard capsules, where gelatin is the shell former, it has been replaced with hydroxypropyl methyl cellulose. In freeze-dried ODTs, the latest trends tend to replace it with methylcellulose (Methocel), certain grades of polyvidone (Kollicoat) and polyethylene oxide [68].

### 4.2. Xanthan Gum

Xanthan gum is a natural polysaccharide, obtained via the fermentation of glucose by the bacterium *Xanthomonas campestris*, with multiple applications mainly as a viscosity enhancer in the formulation of oral or topical pharmaceutical dosage forms [63]. In the lyophilization process of ODTs, it has been used from the beginning in the formulation of Lyoc tablets, playing the role of main matrix former, providing porosity and improved strength, compared to gelatin [1,2]. Xanthan gum is a good suspending agent and viscosity modulator, preventing particle sedimentation in the suspension-type formulations before freezing and insuring dose uniformity [21,43,69,70].

### 4.3. Hydroxypropyl Methyl Cellulose (HPMC)

Hydroxypropyl methyl cellulose has been used as a binder for oral lyophilisates as an alternative to gelatin. The low viscosity grades of HPMC used with success are HPMC E5 (in ODTs) and HPMC E15 (mainly in ODFs), with a viscosity of 5 mPa.s and 15 mPa.s (Methocel E15), respectively [71,72,73,74,75]. HPMC K4 M is a medium viscosity grade of hydroxypropyl methyl cellulose (4000 cPs at a concentration of 2%) and has a lower gelation temperature compared to the grades with higher viscosity [63]. It is a good matrix former for ODFs in combination with glycerin as a plasticizer [76]. In the freeze-drying process of ODTs, HPMC is mixed with polyols and saccharides, in order to optimize the tablet strength and shorten the disintegration time. It has been reported to make solid dispersions with low-soluble drugs, due to the formation of an amorphous mixture. Solid dispersions of HPMC/tenoxicam in different ratios have been reported to increase drug solubility upon lyophilization up to eight fold (for the tenoxicam/HPMC 1:2 ratio) [77].

### 4.4. Methylcellulose

Methylcellulose is widely used in the formulation of oral or topical dosage forms [63]. In ODTs, it acts as a matrix former, with good results in regards of tablet strength, while maintaining short disintegration times. The low viscosity grades can be used in the lyophilization process in concentrations of 2%, providing high porosity and uniform particle size dispersion [78,79,80]. In the investigation of cellulose derivative polymers in formulating lyophilized dry emulsions (LDEs), one of the studies had hydrochlorothiazide as a model drug. Methylcellulose was used as an emulsifier-binder for the obtaining of an oil-in-water LDE, in combination with maltodextrin, with good results [54].

### 4.5. Mannitol

Mannitol has been used in the lyophilization of ODTs for its excellent filler properties, serving as bulking agent/porosity excipient. It has good water solubility and a sweetening capacity of 50% of that of saccharose. An excellent advantage of mannitol in ODTs is its mouthfeel, leaving a cooling sensation, due to its negative heat of dissolution [81,82,83,84]. Unlike sorbitol, it is not hygroscopic, and is also chemically inert and stable in heating conditions [85]. In orodispersible tablets, it is used in concentrations between 10 and 80%, with increasing fracturability. In this matter, mannitol has proven to be more effective than sucrose or sorbitol, which are lyophilized tablets with the same respective polyol concentrations displaying higher hardness [64].

### 4.6. Maltodextrin

Maltodextrin is a non-sweet saccharide mixture of polymers that consists of D-glucose units, prepared by controlled starch hydrolysis [63]. It has been used in tablet technology as binder in wet granulation or filler-binder in direct compression. The grades that have a high dextrose equivalent number (DE) are used in the composition of chewable tablets or lozenges [70,86,87]. In ODTs, it acts as matrix former mixed with cellulose polymers [88], with an increase in maltodextrin concentration resulting in tablets with higher strength [89]. Also, research studies showed that maltodextrin formulations with a high DE value (DE38) showed a faster disintegration time compared to the ones with a lower DE (DE12 or DE24) [54].

### 4.7. Tamarind Seed Gum

Tamarind seed gum (also known as tamarind) is a natural, nonionic water-soluble gum, extracted from the endosperm of the tamarind seed, which contains polysaccharides such as xyloglucan. Primarily used as a thickener in food, in the past years, its use has expanded in cosmetics (creams, lotions) and in pharmaceutical formulations, due to its viscous and adhesive properties, as a binder for tablets (through direct compression and wet granulation). It is biodegradable, non-toxic, and non-irritant; also, it is considered a green ingredient, being obtained from the waste products in the tamarind fruit pulp industry [90,91]. Certain treatments, such as carboxymethylation, enhance its solubility, and its carboxymethylated grade has been used successfully to manufacture ODFs by the 2D-printing technique [91].

A study on lyophilized ODTs containing sodium diclofenac was reported with crude and modified tamarind seed gum as matrix formers, comparative with bulking agents as mannitol/sorbitol/xylitol. It was concluded that crude tamarind gum as a matrix former (~45%) can withstand the lyophilization method and results in ODTs with acceptable properties (in vitro and in vivo disintegration time of approx. 90 s and complete drug release in the first 10 min). An interesting observation was the behavior of bulking agents, with the tablets having different disintegration times depending on the polyol used. In this regard, tamarind/mannitol combination showed best ODT characteristics, whereas the tablets with the combination tamarind/sorbitol and tamarind/xylitol displayed disintegration times over 3 min [90].

This gum is still being studied to make a transition from oromucosal dosage forms to orodispersible tablets.

### 4.8. Powdered Plant Seed Mucilages (Ocimum sp., Plantago ovata, Senna tora)

*Ocimum gratissimum*, *Ocimum sanctum* and *Ocimum basilicum* seeds have found application as pharmaceutical excipients. The seeds can be processed by various treatments (milling, swelling/incubation) and the mucilage can be used as a binder in tablet formulation [92,93,94]. Recently, they have been reported as a good disintegrating agent in ODTs prepared by wet granulation followed by compression [94,95]. *Ocimum sanctum* seeds powder is considered a natural superdisintegrant through wicking action, which was tested in ODTs with nimesulide prepared by direct compression, with good results [96]. Another study published by Nayakal et al. uses *Ocimum basilicum* defatted seed powder as disintegrant in direct compression method for obtaining clopidogrel ODTs, in proportions of 3, 6 and 9% with good results for all formulations, with disintegration times less than 1 min in all cases (32 s for the 9% proportion), and 99% drug release after 10 min) [97]. These studies encourage the use of *Ocimum* sp. seed powder also in lyophilization, given that lyophilization is already a drying technique used for obtaining powdered mucilage, and would maintain its porosity over the freezing/drying states; moreover, its near-instantaneous dissolution upon contact with saliva makes it recommendable as compared to other semi-synthetic or synthetic superdisintegrants.

*Plantago ovata* and *Senna tora* (previously known as *Cassia tora*) mucilage powder are other plant derivative superdisintegrants used successfully in the formulation of ODTs by direct compression, with perspectives to be used also in the lyophilization method [92,93,98].

### 4.9. Pregelatinized Hydroxypropyl Pea Starch

Pea starch is extracted from various pea varieties (*Pisum sativum*), by removing protein and fibers. The pregelatinized hydroxypropyl grade is physically and chemically processed by the etherification of starch with propylene oxide, followed by mechanically processing in water and drying. Thus, the addition of hydroxypropyl groups makes it stable upon cooling without the phenomenon of gelation, which occurs to the other starch grades [99,100]. The pharmaceutical grade Lycoat RS720 contains 35% amylose and 65% amylopectin, is soluble in cold water and forms good mechanical strength, as well as flexible films. In the pharmaceutical field, it has been proposed as an alternative to gelatin capsule shells, in hard and soft capsules, and as a binder in wet granulation, as a natural replacement of synthetic binders [99].

In orally disintegrating tablets, it has been shown to increase tablet strength and provide a short disintegration time. A study published by Mahajan and Kelkar reported a concentration of 1% Lycoat RS720 as a good matrix former in combination with mannitol (0.5%) in lyophilized tadalafil ODTs, with good results for disintegration time (16.6 ± 0.8 s in vitro, 23 ± 2.5 s in vivo), drug release (more than 70% of tadalafil dissolved within 60 s) and stability over 3 months [101]. Also, compared to other starch derivatives, it has little tendency to absorb moisture, thus maintaining tablet integrity.

### 4.10. Polyethylene Oxide (Polyox™ Grades)

Polyethylene oxide (PEO) is a nonionic homopolymer of ethylene oxide and is represented by the formula (OCH2CH2)n, in which n represents the average number of oxyethylene groups. Since its discovery in the 1950s, polyethylene oxide (Polyox™) grades have been used various areas such as agriculture, paper industry, electrode and battery technologies, but they only began to be integrated as pharmaceutical excipients in the 1980s [102,103].

Due to its high molecular mass, as well as nonionic and hydrophilic properties, PEO has been used in the past decades in the formulation of solid dosage forms, especially as modified release systems, such as osmotic pump delivery tablets, gastroretentive dosage forms, and controlled release matrix formulations. The hydrogel layer forming upon hydration of polyethylene oxide provides drug release with various models. In the past years, Polyox grades have been included in the formulation of mucoadhesive dosage forms (buccal films, wafers) and ophthalmic hydrogels (combined with high viscosity grades of HPMC) [102,103,104]. Also, hydrogels containing polyethylene oxide have been prepared in combination with other hydrophilic polymers (carbopol, carrageenan, sodium alginate) to promote chronic wound healing [105].

In the freeze-drying process, Polyox has demonstrated good bulking properties, stabilizing the matrix during the sublimation stage and preventing the collapse of the tablet structure. In orodispersible dosage forms, it has the advantage of high hydrophilicity, fast water absorption and rapid disintegration in the oral cavity. ODFs containing quetiapine formulated with various grades of Polyox (in concentrations of 1–4%, with an optimum of 2%) have demonstrated good properties of tensile strength, a disintegration time less than 30 s, drug release over 80% and good stability [68]. ODFs with apixaban have been formulated for patients with dysphagia, based on a combination of Polyox (20%) and HPMC K4M and prepared via solvent casting technique; the films showed disintegration times less than 30 s, good tensile strength properties, good stability over a 6 months period and bioequivalence parameters compared to the original apixaban-marketed formulation [106]. In ODTs, low-viscosity Polyox grades (Polyon N10) were proven suitable as binders in 10% concentration, in combination with mannitol, resulting in robust ODTs with disintegration times below 40 s [107].

### 4.11. Side-Stream Lactose

This is a sustainable type of lactose obtained from byproducts from the dairy industry, like whey permeate and lactose mother liquor, and is nowadays increasingly valorized for pharmaceutical tablet manufacturing and nutrient-rich growth media. It has been proposed to be used as an excipient in spray-drying and freeze-drying, but also for direct compression mixed with other excipients such as microcrystalline cellulose [108].

### 4.12. Polyvinyl Alcohol

Polyvinyl alcohol (PVA) has been proposed as an alternative binder to gelatin in lyophilized ODTs and was found to be suitable for both low-dose and high-dose drugs. Vanbillemont et al. tested the characteristics of paracetamol high-dose ODTs and concluded that the optimal proportion is 2.76% PVA. Also, for low-dose ODTs containing hydrochlorothiazide, a proportion of 2.96% PVA was found to have minimum probability of failure, according to the experimental design [43].

### 4.13. Co-Processed Microcrystalline Cellulose Grades

Microcrystalline cellulose is an excellent excipient for direct compression, acting as a filler-binder, but it can also be used as a binder in other techniques such as wet granulation, dry granulation, extrusion, pelletization. It also possesses lubricant properties and acts as a disintegrant in tablets through wicking action [63,109]. Depending on their particle size, microcrystalline cellulose grades differ in their flowability, moisture uptake and are recommended for different manufacturing technologies. The *Avicel CE15* grade is a co-processed excipient composed of microcrystalline cellulose (85%) and guar gum (15%), specially designed for buccal dosage forms (e.g., chewable tablets) to improve palatability [63].

In ODTs, it has been explored for direct compression formulations or dry granulation (slugging), in combination with other microcrystalline cellulose grades [110,111,112,113,114]. Also, *Avicel CE15* has been used in combination with mannitol in the lyophilization process, with the obtained ODTs showing good porosity and short disintegration times (<10 s) [115].

Avicel HFE102 is a co-processed grade of co-spray dried microcrystalline cellulose (90%) and mannitol (10%), with a small particle size of approx. 100 µm [110,111]. It has better flowability, compaction and disintegration properties compared to traditional microcrystalline cellulose grades; at the same time, it displays less sensitivity to lubrication with stearates. It has been recommended for its good sensory attributes, to be used in chewable tablets, but recent studies have shown good applications also in ODTs [112].

### 4.14. Gellan Gum

Gellan gum is a natural anionic polysaccharide, produced through fermentation by the *Sphingomonas* spp. bacteria, which were isolated as *Sphingomonas elodea* in the U.S.A. from the lily plants that grew in the ponds. It was first approved as a food additive in Japan in 1988, being subsequently introduced as a pharmaceutical excipient and additive. It is a water-soluble, off-white powder, with a chemical composition of repeating units of D-glucose (~60%), L-rhamnose (~20%) and D-glucuronic acid (~20%) units. Its aqueous solutions are viscous at low concentrations, forming clear thermoreversible gels, which are less pH-dependent compared to other colloids [116,117,118]. In the pharmaceutical field, it has been used as a binder in modified release tablets, also in nanotechnology (drug-loaded gellan beads), ocular drug delivery and the formulation of hydrogels and thin films [118,119].

In orodispersible tablets, gellan gum has been implemented recently as a matrix former and as a binder with taste masking properties. One example of published study involves the preparation of atomoxetine ODTs by wet granulation, with various proportions of gellan gum (19–38%). Best results regarding in vitro disintegration time and drug release were recorded for the lowest concentrations of gellan gum, which also contributes to tablet disintegration, due to its swelling properties [120,121].

For the ODTs prepared by lyophilization, gellan gum has been used as a matrix former for drug-loaded nanoparticles, in order to prepare the dispersion to be freeze-dried [60]. Proportions of 15–45% of gellan gum were used in combination with mannitol in formulating freeze-dried ODTs with silymarin, with a notable in vitro disintegration time below 30 s. Increasing gellan gum ratio leads to higher tablet resistance, but with longer disintegration times [122].

### 4.15. Guar Gum

Guar gum is a natural polysaccharide derived from *Cyanopsis tetragonolobus* sp., highly used in pharmaceutical formulations for its viscosity properties [63]. Guar gum has been used successfully in the formulation of fast melt films prepared by various methods, including freeze-drying, due to its characteristics of rapid disintegration [123]. In orodispersible tablets, it has been used as a tablet matrix, making porous structures that disintegrate rapidly. However, a limit proportion should be used due to its swelling behavior, given that high concentrations (more than 8%), lead to a thick gel layer surrounding the tablet that may delay disintegration [124].

Modified guar gum (esterification through nucleophilic substitution with 2-Dodecen-1-yl succinic anhydride) modulates its swelling capacity and improves its dispersity in water, thus making it more adequate for rapid dispersion. The modified grade was formulated in ODTs with the model drug metoclopramide, with the tablets showing good pharmaco-technical and release pattern characteristics (more than 95% in the first 5 min) [125].

### 4.16. Amino Acids

Amino acids are a new class of excipients that are explored currently in the formulation of zero saccharide/polyols oral lyophilisates, with the aim of replacing the conventional matrix formers and to optimize the parameters of the lyophilization cycles. However, amino acids vary in their behavior and properties: the low glass transition temperature of some amino acids leads to the collapse of the matrix upon lyophilization, while other amino acids lack proper wettability, which is needed for providing short disintegration time [62,126].

A combination of proline and serine was reported by Al Husban et al., improving the qualities of lyophilized ODTs by choosing an adequate ratio of the two amino acids (45:55 proline/serine), for good wettability and adequate hardness [45]. Recently, lyophilized ODTs were formulated with alanine and serine with good results for high serine concentrations; combining it with alanine also increases the wettability of the ODTs [126]. Another study reports a combination of three amino acids (L-arginine, L-lysine, L-histidine 1.%, respectively) for obtaining freeze-dried ODTs with the antipsychotic drug lurasidone, with the aim of increasing drug solubility and absorption through the buccal epithelium (Table 5) [62]. The amino acids, especially L-histidine, prevented drug aggregation during freeze-drying and increased content uniformity. Also, it was observed that the presence of the amino acids L-arginine and L-lysine increases the hardness of the ODTs, while L-histidine and L-arginine accelerated transmucosal absorption [62].

### 4.17. Chitosan Grades and Chitin

Chitosan is a natural cationic, biodegradable polysaccharide extensively used in the past decades in the formulation of various dosage forms, due to its unique properties. In ODT formulations, its disintegrating, mucoadhesive and dissolution-enhancing characteristics have recommended chitosan as a multi-functioning excipient [127]. Medium molecular weight chitosan has been tried as superdisintergant, working by capillary action in the formulation of orodispersible tablets [128].

ODTs with meloxicam prepared by the wet granulation method with 7% chitosan as disintegrant displayed acceptable properties only at certain compression forces (10.8–11 kN) [128,129]. A combination of chitosan and alginate (1:1) was used as a disintegrating system in ODTs containing the highly water-soluble drug metoclopramide, prepared by the compression method. An interesting point was the addition of chitin (5–10%), which enhanced porosity and decreased tablet disintegration time [130,131]. Chitosan demonstrated good disintegrating properties at low concentrations (3.5–7%), being studied in ODTs with aspirin prepared by direct compression. These low concentrations are relevant, since it prevents jellification, which delays tablet disintegration [129].

Recently, chitosan nanoparticles have been implemented for the SNELT formulation of low-soluble drugs, such as rosuvastatine, which is a BCS class II drug (low solubility, high permeability). The drug was loaded into phospholipid nanoparticles coated with chitosan (chitosomes), which were further formulated for lyophilization, with the addition of matrix former excipients [132] (Table 5). The resulting ODTs had very short in vitro and in vivo disintegrating time and overall enhanced pharmacokinetic parameters compared to the non-loaded rosuvastatine lyophilized ODTs (Table 5) [132].

### 4.18. Eudragit E Grades

Eudragit E grades are cationic copolymers based on dimethylaminoethyl methacrylates and neutral methacrylates (such as buthyl and methyl methacrylate), which are soluble in biological fluid below pH = 5, and are therefore used for immediate release coatings of microparticles, tablets, minitablets, in order to achieve taste masking, protection from moisture and light or the bioavailability-enhancing characteristic of low-soluble drugs in the gastric juice. It is available in three grades: Eudragit E100 (granules), Eudragit EPO (powder) and Eudragit E 12.5 (organic solution). Eudragit E100 and EPO grades have been used in orally disintegrating tablets for coating the drug particles with the purpose of taste masking, because they are not soluble in the pH of saliva, thus keeping the coated units intact [133,134].

Granulation of the Eudragit E100 powder with tramadol in the proportion of 1:1, followed by the mass extrusion method was used to obtain taste-masked granules which were further compressed [135]. The EPO coating suspension (35% optimal amount) has been used for taste masking clindamycin hydrochloride beads for the preparation of ODTs via direct compression, using a fluid bed system. The taste masking efficiency was demonstrated by maintaining coating integrity, with good results [136].

Eudragit EPO has also been used in the lyophilization method. In a study reported by Bhoyar et al., trimetazidine hydrochloride particles were coated with Eudragit EPO by a physical mixture in an optimal proportion of 1:3, respectively, followed by lyophilization to obtain ODTs. The coating efficiency was tested by drug release in salivary fluid, demonstrating the capacity of taste masking of the prepared granules (Table 5) [137].

**Table 5:** Examples of formulation studies for lyophilized ODTs.

API	Excipients	In Vitro Disintegration Time	Type of Lyophilized Dispersion
Hydrochlorothiazide (low dose) Paracetamol (high dose) [43]	mannitol, polyvinyl alcohol/Xanthan gum	2.1 ± 2 s/ 3.9 ± 1.5 s 3–12 s	Dispersion
Terbutaline sulphate [67]	gelatin, sodium alginate, PEG 4000, Pluronic F68, HPMC, simethicone	11 s	Solution
Sumatriptan [69]	gelatin, Plasdone K90D, mannitol, sucrose, glycine, Xanthan gum, sucralose, magnesium stearate, disodium EDTA, menthol, camphor	12.5 ± 3.1 s	Dispersion
Diclofenac potassium [70]	gelatin, mannitol, lactose, maltodextrin, glycine, potassium chloride, sodium chloride, hydroxypropyl cellulose, Xanthan gum	23 ± 0.9 s	Solution
Nimesulide [71]	maltodextrin, Methocel E15	<10 s	Aqueous dispersion
Celecoxib [72]	HPMC E33, PVP K30, HPC-SSL, PEG 4000, trehalose, poloxamer 188, CMC-Na, mannitol, sorbitol	4–8 s	Dispersion
Aprepitant [73]	HPMC E5, sucrose, Tween 80, Poloxam 188, mannitol, HPC-SL, pullulan	<5 s	Dispersion
Cinnarizine [74]	mannitol, hydroxypropyl methylcellulose (HPMC E5), glycine	27.5–56 s	Aqueous solution
Olmesartan medoxomil [75]	gelatin/sodium alginate/HPMC, Na-CMC, mannitol, glycine, Soluplus	32 s	Dispersion
Naratriptan-Naproxen [78]	gelatin, sucrose, sodium alginate, methylcellulose, hydroxyethylstarch	<10 s	Dispersion
Naproxen [79]	sucrose, hydroxyethyl starch, Poloxamer 188, menthol flavor	2.7 ± 3 s	Dispersion
Meloxicam [80]	methylcellulose, mannitol	<10 s	Dispersion
Isosorbide dinitrate [81]	gelatin, mannitol, glycerin, tween/polyethylene glycol	<60 s	Aqueous dispersion
Cannabis extract [82]	gelatin, mannitol, tween 80, sucralose, sodium methylparaben, sodium propylparaben	<10 s	Solution
Deferasirox [83]	gelatin, mannitol, sodium alginate, PEG 4000, Pluronic F-68, HPMC, simethicone	5 s	Dispersion
Acemetacin [84]	gelatin, polyvynyl pyrrolidone K90, glycine, mannitol	26.6 s	Nanosuspension
Ketoprofen [85]	gelatin, glycine, sorbitol	<10 s	Dispersion
Fexofenadine [87]	gelatin, maltodextrin, acacia, glycine	<10 s	Dispersion
Diclofenac sodium [90]	Tamarind seed gum, mannitol/sorbitol/xylitol, PEG 4000	<90 s	Solution
Tadalafil [101]	pregelatinized hydroxypropyl pea starch (Lycoat RS720), mannitol, magnesium stearate, flavor	16.6 ± 0.8 s	Dispersion
Lurasidone [62]	gelatin, mannitol, L-histidine, L-arginine, L-lisine, Polysorbate 80, Triton X100	<120 s	Dispersion
Chlorpheniramine maleate [114]	mannitol, gelatin, microcystalline cellulose, glycine, Tween 80, PVPK30, PEG 6000, PEG 4000	20 s	Dispersion
Acetaminophen- Pregabalin [115]	gelatin, Avicel CE15/HPMC, mannitol	<10 s	Dispersion
Silymarin [122]	mannitol, gellan gum/pullulan/alginic acid glycine	28 ± 0.33 s	Dispersion
Trimetazidine [137]	gelatin, Eudragit EPO, glycine, mannitol	12 s	Dispersion
Hydrochlorothiazide [54]	maltodextrin, methylcellulose/HPMC, Mygliol, potato starch	<10 s	LDE tablets
Vitamin K [56]	gelatin, Avicel PH-101, fumed silica, glycine, mannitol, lactose, HPMC 4000Cp		SNELTs
Febuxostat [59]	gelatin, xylitol, mannitol, lactose, croscarmellose sodium, HPMC	<180 s	SNELTs
Finasteride [55]	gelatin, mannitol, HPMC, Avicel, silica, Plasdone XL	6.49–11.24 min	SNELTs
Piroxicam [21]	Poloxamer 188, maltodextrin (DE39), mannitol, PEG 4000, gelatin/Xanthan gum/croscarmellose sodium, aspartame	<10 s	SNELTs
Rivaroxaban [60]	sodium alginate, gellan gum/hydroxypropyl methylcellulose, mannitol	118 s	SNELTs (freeze-dried NMMs)
Rosuvastatine [132]	(phospholipid, tween 80, chitosan), pullulan, HPMC, aspartame, xylitol, Plasdone XL	2.1 ± 0.17 s	SNELTS (freeze-dried chitosomes)
Zaleplon–Lavender oil [57]	gelatin, mannitol, Avicel PH-101, Kryon T-314, fumed silica, sodium carboxymethyl cellulose	<30 s	SNELTs
Meloxicam–Peppermint oil [58]	gelatin, fumed silica, hydroxypropyl cellulose, spray dried lactose, sorbitol	3 ± 1 s	SNELCs

## 5. Emerging Perspectives—Hybrid Technologies and Future Platforms

### 5.1. Lyophilization and 3D-Printing

3D-printing technologies have emerged and developed rapidly over the past three decades, evolving from prototypes to industrial manufacturing [138,139]. Their application in pharmaceutical dosage forms has especially found a place in tablet manufacturing, involving layer-by-layer material deposition, through the use of a digitally controlled machine. The main advantage of implementing 3D printing is tailoring the dosage regimen to the patient needs, allowing the combination of drugs and improving patient adherence [138,140].

3D-printing technologies are especially useful in developing ODTs, since the resulting porous structure lacks the necessity of disintegrant addition. Some of the 3D-printing technologies that have been implemented for this purpose are as follows: (a) binder jetting (BJ), which involves pouring a liquid onto a powder bed; (b) semi-solid extrusion (SSE), which uses the extrusion of drug-loaded pastes to create *printlets*; (c) direct powder extrusion (DPE), which uses the direct processing of powder onto the dosage forms, without the need for filament production; (d) fused deposition modeling (FDM), which uses hot melt extrusion, based on melting the thermoplastic filaments containing the drug, is used mainly for modified release, but has been lately adapted to ODTs; (e) selective laser sintering (SLS), which is based on directly sintering the deposited powder materials without solvents, by means of a laser, thus creating various geometries for the ODTs [139,140].

**Figure 2:**
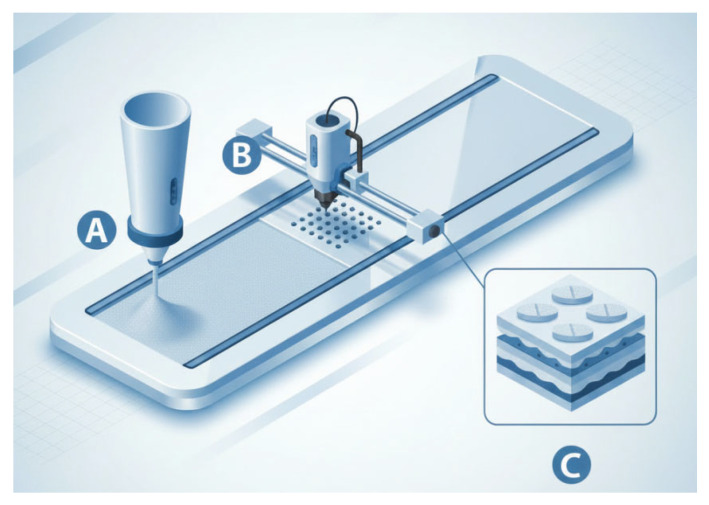
BJ printing for obtaining OTDs. (**A**) Powder mixture is spread as a uniform layer to build the platform. (**B**) The binder solution is sprayed on the powdered blend. (**C**) Fusioning binder solution/powder layer by layer until the final tablet is formed. (Used Ai-Claude Opus 4.5).

Out of all the methods, BJ printing has received approval from the FDA, with the first marketed tablet product prepared by this method, containing the drug levetiracetam (Spritam^®^) [141,142]. This technique implemented on an industrial scale was patented by the Aprecia Pharmaceuticals Company under the name ZipDose^®^ Technology, as a platform that can be adapted to developing and industrially manufacturing tablets that disintegrate very fast, including ODTs (Figure 2). The process is set up on a large scale, with GMP quality, batch-to-batch reproducibility and a special manufacturing machine called M0 [143,144].

**Figure 3:**
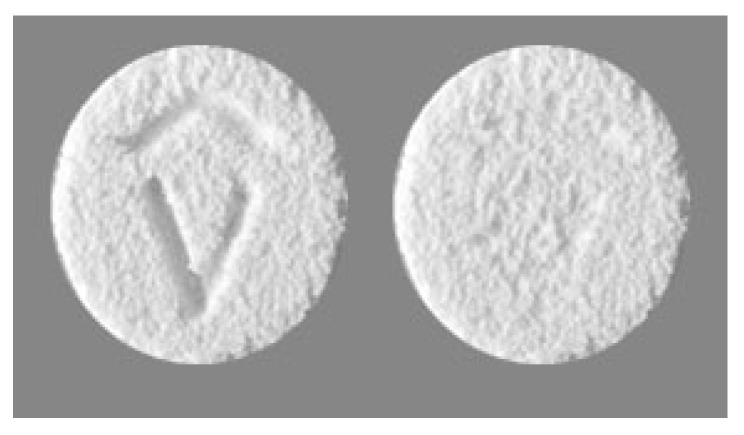
The porous appearance of Spritam^®^ levetiracetam tablets (https://www.drugs.com/dosage/spritam.html, accessed on 24 April 2026).

The limitations of the process are low manufacturing speed (approx. 100 tablets/hour), challenges concerning taste masking (allowing it only with sweeteners and flavors) and limitations in the drug doses. However, the Zipdose^®^ levetiracetam by Aprecia is available in doses of 250 mg, 500 mg and 1000 mg, commercialized as tablets for oral suspension (Figure 3). The technology allows engraving a logo on one side of the tablet and requires packaging in peel-off blister packs. This technology is increasingly being utilized for developing ODTs with other drugs in various doses [143,144].

Combining 3D-printing (especially BJ printing) with lyophilization is a promising technological frontier in pharmaceuticals. Studies have been conducted with 3D-printed sodium carboxymethyl cellulose hydrogels (through FDM printing) loaded with food colorant as model substance and further put to lyophilization. It was concluded that lyophilization modified the colorant release kinetics, also prolonging hydrogel dissolvability [145].

In ODTs, this hybrid technique enables the creation of structures with extremely high internal porosity, leading to a disintegration time of less than 10 s, while maintaining structural integrity. Integrating lyophilization with additive manufacturing allows for on-demand production of patient-specific doses with an optimized drug release profile. However, combining these two processes addresses individual limitations, such as the poor mechanical strength of freeze-dried tablets or the thermal instability risks of certain 3D-printing methods. Binder jet-printing at room temperature followed by lyophilization protects sensitive drugs from degradation. Incorporating emulsions or liquid-based carriers into a 3D-printed structure that is further freeze-dried can increase the bioavailability of poorly soluble drugs. Recent studies have been reported in the literature, combining freeze-drying with new ink jet-printing technologies. This was done for carvedilol (a BCS class 2 drug) by ink printing the drug dispersion (ink) over freeze-dried HPMC gels, obtaining modular units. Carvedilol exhibited different release results, depending on the HPMC grade and content (3 to 10% in the lyophilized modules) [146].

### 5.2. Lyophilization and Mucoadhesion

These two techniques may seem at first glance opposite in terms of drug release and a mismatch in the orodispersible tablet concept. However, the need for transbuccal absorption in some areas like dentistry has made this combination possible, given the combined qualities of the two technologies: the resulting ODTs are designed to instantly disintegrate in the mouth upon contact with saliva, while adhering to the oral mucosa to improve local or systemic drug absorption.

However, challenges related to these hybrids include disintegration, since the formation of thin gel layer around the tablet could prolong disintegration time; also, tablet strength remains a concern, given the specifics of the lyophilization technique [147,148,149]. Local anesthetics like lidocaine and prilocaine were formulated in ODTs with pullulan and HPMC as mucoadhesive polymers; the drugs showed increased permeation through the buccal epithelium. The formulation involved combining ODT matrix formers (gelatin, mannitol, glycine) with mucoadhesive polymers and permeation enhancers [147].

Currently, there are few studies on combining these two directions; however, this topic is emerging for various substances that need prevention from degradation (provided by lyophilization) and mucoadhesion for buccal absorption: probiotics, enzymes, and peptides.

### 5.3. Lyophilization and Electrospinning

Electrospinning has been used for several years in obtaining nanofibers with medical applications for various administration routes: oral, buccal, rectal, vaginal, dermal, ocular [150,151]. For orally disintegrating dosage forms, it has been performed successfully for ODFs with drugs such as aripiprazole [152], isoniazid [153], telmisartan [154], amlodipine besylate [155], and rosuvastatin calcium [155]. For ODTs, this technique is used to produce nanofibers having high surface-to-volume ratio, and very fast disintegration (within seconds) upon contact with saliva, providing a non-gritty sensation and pleasant mouthfeel. In the literature, these structures are yet to be optimized into ODTs, since they are called ‘orodispersible nanofibers’ or ‘fast dissolving electrospun mats’. Studies have been done for diclofenac [156], meloxicam [157], and donepezil [158]. The molecular dispersion of the drug throughout the electrospun nanofibers can provide increased bioavailability; also, compared to the lyophilization process, which requires special conditions, electrospinning reduces costs, being done at room temperature.

The hybrid technique combining electrospinning and lyophilization is an emerging pharmaceutical strategy used to create next-generation dosage forms. It has been used in tissue engineering for generating hybrid 3D scaffolds for wound healing, with better results than the scaffolds obtained through lyophilization alone (decreased pore diameter, optimized swelling behavior) [159]. Other approaches include the fabrication of electrospun scaffolds for vascular tissue engineering, followed by the addition of a lyophilized layer of polymer (gelatin), to enhance certain characteristics regarding vascular muscle penetration [160].

In ODT manufacturing, this hybrid approach would integrate the high surface area of nanofibers with the highly porous, structural stability of a lyophilized matrix to overcome certain ODT limitations, such as high friability or decreased bioavailability for certain APIs. Additionally, it can provide enhanced protection for sensitive drugs and optimize energy consumption.

## 6. Conclusions

Modern dosage forms require the implementation of new techniques into old patterns of formulation and manufacturing pathways. The lyopilization process provides transition from the simple concept of rapid drug release to advanced solubilization and transbuccal absorption through the new SNELT structures. New excipients have been implemented, in order to overcome the limitations of traditional ones; these can be either new processed grades, or green, sustainable ingredients, which are side-stream or plant-derived.

The use of lyophilized ODTs may serve as a promising strategy for developing therapeutic protein delivery platforms, given that biological therapies often preclude parenteral delivery due to high intrinsic risks. Thus, the freeze-dried SNELTs provide extended drug stability, taste masking, and transbuccal absorption with increased bioavailability. All these new approaches merge into future hybrid platforms which will integrate lyophilization with 3D printing, mucoadhesion, electrospinning or other approaches or techniques, in order to obtain more effective, personalized ODTs.

## Figures and Tables

**Table 1:** Advantages vs. limitations of the lyophilization method for ODT preparation [45,46].

Advantages	Limitations
Very short disintegration times High porosity of the final product	Low friability
	Low hardness (fracturability), compared to compressed ODTs
Possible solubility and bioavailability enhancing by use of various techniques (e.g., SNELTs) Good batch-to-batch uniformity	Critical process control parameters
	Need for peel-off blister packaging
Possibilities of preserving heat-sensitive APIs Possibilities of taste masking	Expensive, time-consuming

**Table 3:** Comparative analysis between the main lyophilization techniques for manufacturing ODTs [1,3,8,9,46,50,51,52,53,54,55,56,57,58,59].

Feature	Lyoc	Zydis	Pharmafreeze	Quicksolv	SNELTs
Manufacturing complexity	Moderate–High Preparation of a O/W emulsion	High Multi-step lyophilization process directly in blisters	High Lyophilization process requires specialized low temperature control	Moderate–High Two-step solvent extraction/ lyophilization process	Moderate Optimized lyophilization Specific matrix-forming polymers
Drug loading capacity	Moderate–High ≤500 mg in Lyoc, ≤700 mg in Lyoc–Sorbet	Low for soluble APIs, (≤60 mg) Moderate–High for insoluble APIs (≤400 mg—Zydis Ultra)	Low–Moderate ≤250 mg	Low–Moderate	Moderate–High APIs loaded in nano-carriers
Disintegration time	Fast <10 s	Ultra-Fast <3 s	Fast <15 s	Fast 5–10 s	Fast <10 s
Mechanical Strength	Acceptable	Poor	Good	Good	Acceptable
Taste masking	Good High filler content-mannitol/lactose	Excellent Ideal though for tasteless drugs; bitter APIs require Zydis Ultra-Coating Technology	Good Sugar-based excipients	Moderate Addition of sweeteners/ Flavors	Excellent Drug particles coated in nanostructures
Stability (shelf life)	Good	Excellent	Excellent	Good	Good
Packaging	Standard/ Peel-off blisters	Peel-off Blisters	Standard	Standard/Alu-Alu blisters	Standard/Alu-Alu blisters
Scale-up feasibility	Good (but expensive)	Acceptable (due to the complex formulation)	Acceptable (slow, batch process)	Challenging (safety and environmental issues due to solvent extraction stages)	Good Designed for better process efficiency
Costs	Moderate–High filler content and long drying time	High Specialized equipment/blister packaging	High Energy-intensive primary drying	High Time-consuming lyophilization, solvent handling stages	Moderate–High

**Table 4:** Traditional and emerging excipients used as matrix formers/bulking agents in freeze-dried ODTs.

Matrix Formers	Bulking Agents
Traditional excipients	
Gelatin Acacia, Tragacanth, Xanthan gum Carrageenan Pectin Alginates Cellulose derivatives (HPMC, MC, CMC-Na)	Mannitol, Sorbitol, Xylitol Lactose Maltodextrin
Emerging excipients	
Tamarind seed gum Pregelatinized Hydroxypropyl Pea Starch Powdered lant seed mucilages (*Ocimum seed* sp., *Plantago ovata*, *Senna tora*) Gellan gum Guar gum Povidone Co-processed microcrystalline cellulose grades Polyvinyl alcohol (PVA) Chitosan/Chitin Polyox grades Eudragit E grades	Side-stream lactose Amino acid combinations: Proline/Serine L-arginine/L-lysine/L-histidine Alanine/Serine Glycine

## Data Availability

The original contributions presented in this study are included in the article. Further inquiries can be directed to the corresponding author.

## Abbreviations

The following abbreviations are used in this manuscript:

ODTs	Orally Disintegrating Tablets
ODFs	Orally Disintegrating Films
SNEDDS	Self-Nanoemulsifying Drug Delivery Systems
SNELTs	Self-Nanoemulsifying Drug Delivery Systems
LDEs	Lyophilized Dry Emulsions
HPMC	Hydroxypropyl Methyl Cellulose
MC	Methyl Cellulose
CMC-Na	Sodium Carboxymethyl Cellulose
BCS	Biopharmaceutical Classification System
BJ	Binder Jetting
